# A security network in PSI photoprotection: regulation of photosynthetic control, NPQ and O_2_ photoreduction by cyclic electron flow

**DOI:** 10.3389/fpls.2015.00875

**Published:** 2015-10-15

**Authors:** Frédéric Chaux, Gilles Peltier, Xenie Johnson

**Affiliations:** ^1^CEA, Direction des Sciences du Vivant, Institut de Biologie Environnementale et de Biotechnologie, Laboratoire de Bioénergétique et Biotechnologie des Bactéries et Microalgues, CEA Cadarache, Saint-Paul-lez-Durance, France; ^2^UMR Biologie Végétale et Microbiologie Environnementale, Centre National de la Recherche Scientifique, Saint-Paul-lez-Durance, France; ^3^Laboratoire de Bioénergétique et Biotechnologie des Bactéries et Microalgues, Aix Marseille Université, Saint-Paul-lez-Durance, France

**Keywords:** cyclic electron flow, PSI photoinhibition, non-photochemical quenching, oxygen photoreduction, luminal acidification, photosynthetic control, ATP, malate valve

## Abstract

Cyclic electron flow (CEF) around PSI regulates acceptor-side limitations and has multiple functions in the green alga, *Chlamydomonas reinhardtii.* Here we draw on recent and historic literature and concentrate on its role in Photosystem I (PSI) photoprotection, outlining causes and consequences of damage to PSI and CEF’s role as an avoidance mechanism. We outline two functions of CEF in PSI photoprotection that are both linked to luminal acidification: firstly, its action on Photosystem II with non-photochemical quenching and photosynthetic control and secondly, its action in poising the stroma to overcome acceptor-side limitation by rebalancing NADPH and ATP ratios for carbon fixation.

In the early years of photosynthesis research, a cyclic photophosphorylation was described that required ferredoxin (Fd), did not evolve oxygen (O_2_) and resulted in the accumulation of ATP (Arnon et al., 1954). From this observation, experiments performed in a variety of organisms from cyanobacteria to higher plants using a combined pharmacological and *in vitro* approach created a robust model for what is now referred to as cyclic electron flow (CEF; thoroughly reviewed, in Bendall and Manasse, 1995). More recently, *Arabidopsis thaliana* lines altered in CEF have been identified and have enriched the ways we have to study these pathways (Joet et al., 2001; Munekage et al., 2002, 2004; DalCorso et al., 2008). Biochemical approaches have shown that the Proton-Gradient Regulator5 (PGR5) and PGR5-Like1 (PGRL1) proteins form an interaction that results in a ferredoxin-plastoquinone reductase (FQR) activity (Hertle et al., 2013). In the unicellular, green alga, *Chlamydomonas reinhardtii*, this pathway and the function of these proteins is conserved (Petroutsos et al., 2009; Tolleter et al., 2011; Johnson et al., 2014). In *Chlamydomonas* a second type of CEF is also in operation where the mediator at the level of the PQ pool is a type-2 NADPH dehydrogenase (Desplats et al., 2009), with the *nda2* mutant shown to have a phenotype in CEF (Jans et al., 2008). Here, we focus on the PGR5 pathway and work done on the *Chlamydomonas* mutants *pgr5* and *pgrl1* mutants, that both demonstrate no PGR5/PGRL1-dependent CEF (Alric, 2014). Our focus is on *Chlamydomonas* but due to the conservation of this pathway we also make reference to work done in other photosynthetic organisms.

Cyclic electron flow is a generator of proton motive force that (i) can produce supplementary ATP to meet ATP:NADPH requirements for the Calvin Benson Bassham (CBB) cycle and the CO_2_ concentrating mechanism (CCM; reviewed by Alric, 2010), and (ii) triggers regulatory mechanisms, namely non-photochemical quenching (NPQ) and cytochrome *b*_6_*f* complex (cyt*b*_6_*f*) “photosynthetic control” (Joliot and Johnson, 2011). Its rate is highest under conditions where the stromal poise is reduced, thus PGR5-CEF has been considered as a regulator of redox homeostasis for the photosynthetic chain (Nishikawa et al., 2012). Among the phenotypes observed in CEF-altered strains of both *Arabidopsis* and *Chlamydomonas*, Photosystem I (PSI) photoinhibition arose in conditions of high light or limiting CO_2_ (Munekage et al., 2002; Dang et al., 2014; Johnson et al., 2014) and fluctuating light (Suorsa et al., 2012) leading to the assignment of yet another role for PGR5-CEF. While Photosystem II (PSII) photoinhibition is frequently observed and has complex models that describe the mechanism (Murata et al., 2012), PSI photoinhibition remains poorly understood. In this work, we review the potential causes of photoinhibition that occur at the acceptor-side of PSI and the processes triggered by CEF that can contain it. For the sake of comprehensive reviewing of mechanisms involved in PSI photoprotection, other connected pathways are also introduced.

## Acceptor-Side Limitation is the Cause of PSI Photoinhibition

**FIGURE 1:**
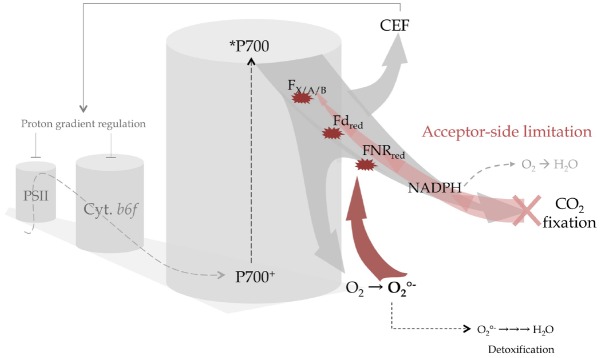
**Acceptor-side limitation and excess electron flow promotes CEF or in its absence leads to the irreversible damage of PSI centers.** The linear electron flow coming from PSII (gray dashed arrow) is the source of electrons for the PSI reaction center (P700^+^/P700) that transfers electrons from the chlorophyll excited state (P700^*^) and subsequently delivers to downstream acceptors within PSI (F_*X*_, F_*A*_, F_*B*_ iron-sulfur centers) then to stromal electron carriers (ferredoxin, FNR, NADP^+^) (light gray arrow). When CO_2_ fixation decreases, acceptor-side limitation gradually leads to accumulation of NADPH and overreduction of stromal and PSI electron carriers (light red arrow). In this case electrons are redirected to O_2_ either at the level of NADPH without the production of reactive oxygen species (ROS; gray dashed arrow) or produce the very reactive superoxide anion radical (${\text{O}}_{\text{2}}^{•-}$) at the level of F_*X*_, F_*A*_, F_*B*_, Fd, and FNR with a rate exceeding the detoxification process. Thus ${\text{O}}_{\text{2}}^{•-}$ will irreversibly destroys the centers (red arrows) resulting in an inability to oxidize *P700 and on a longer time scale the degradation of the entire PSI complex. Preventing this scenario, cyclic electron flow triggers downregulation of linear electron flow at the site of PSII and *cytb*_6_*f* by enhancing proton accumulation in the lumen.

PSI photoinhibition was first reported in isolated chloroplasts submitted to strong light (Jones and Kok, 1966). Satoh was able to differentiate two types of damage that corresponded to damage to the two photosystems using fragmented chloroplasts. Artificial donors were used to measure the capacity of PSI to transfer electrons to terminal acceptor, NADPH. These experiments showed that the addition of the PSII inhibitor, DCMU specifically but incompletely prevented photo-inactivation of PSI (Satoh, 1970a,b). Photo-inactivation of PSI was avoided by addition of excess Fd showing that PSI photoinhibition is an acceptor-side limited phenomenon. The observation that the same group of co-factors that could enhance CEF (including Fd) were also involved in the avoidance of photoinactivation of PSI led to the discussion of CEF as a photoprotectant for PSI (Satoh, 1970c). Further studies demonstrated the destruction of PSI-bound iron-sulfur centers (F_*X*_, F_*A/B*_) by oxidative species primarily superoxide anion radical (${\text{O}}_{\text{2}}^{•-}$) (Sonoike et al., 1995). Production of ${\text{O}}_{\text{2}}^{•-}$ can occur within: the iron-sulfur centers of PSI, reduced Fd and stromal flavodehydrogenases (NADP+ ferredoxin dehydrogenase, glutathione reductase and monohydrate ascorbate reductase) in plant chloroplasts (discussed, in Asada, 2000). In permissive conditions, radicals are enzymatically neutralized into water, resulting in the net uptake of O_2_ reported by Mehler (1951), establishing a pseudo-cyclic pathway for electrons known as the water-water cycle. When radical production exceeds detoxifying capacity, ${\text{O}}_{\text{2}}^{•-}$ irreversibly damages PSI primary acceptors (F_*X*_, F_*A/B*_) and prevents stable accumulation of P700^+^ in high light (Figure 1). This is because of fast charge recombination at the level of intermediary acceptors A_0/1_ (Setif and Brettel, 1990). The resultant decrease in the quantity of oxidizable P700 is thus a common measurement for probing the photoinhibition of PSI.

Interestingly, no singlet oxygen (^1^O_2_) is produced in overexcited PSI (triplet excited state, ^3^P700) because P700 is sterically screened from O_2_ (Setif et al., 1981). Hence, P700^+^ and ^3^P700 are most probably efficient quenchers of excess excitation of plant PSI as observed for cyanobacterial PSI (Schlodder et al., 2005; Shubin et al., 2008). Contrarily, ^1^O_2_ is the main photo-damaging species produced in acceptor-side limited PSII (^3^P680) (Durrant et al., 1990). It is remarkable that O_2_ can be sensitized in PSII and not in PSI (Hideg and Vass, 1995), in other words that ^1^O_2_ production within PSII was not evolutionarily eliminated. Over time this may be why a signaling role has developed for ^1^O_2_ (Telfer, 2014) resulting in some selectivity in the degradation of PSII protein under photoinhibitory conditions. As compared to the “monolithic” architecture of PSI, the modular architecture of PSII allows for a unique degradation of damaged D1 and re-use of other subunits (extensively reviewed, in Caffarri et al., 2014) and may be another reason why PSII damage-and-repair cycle has been a target of selective pressure. On the contrary, PSI has no known molecular mechanism *per se* to set its turnover in tune with light intensity. The protection of PSI from photoinhibition would appear to require a set of distinctly different properties than that of PSII (Allahverdiyeva et al., 2015) which includes buffering acceptor side limitations in the stroma. Selective, irreversible photoinhibition of PSI in *Chlamydomonas* is observed to occur both in CEF-altered strains (Dang et al., 2014; Johnson et al., 2014; Kukuczka et al., 2014; Bergner et al., 2015) and in strains with severe acceptor side limitations such as those lacking RuBisCO (Johnson et al., 2010). *Crpgr5* and *Crpgrl1* strains demonstrate decreased amounts of oxidizable P700 and PSI protein measured by western hybridization after exposition to high light (Johnson et al., 2014; Kukuczka et al., 2014) and after transition from high (2%) to atmospheric concentrations of CO_2_ (Dang et al., 2014). In the following sections we present CEF’s role in triggering several mechanisms avoiding long-lasting limitations at the acceptor-side of PSI.

## CEF Triggers Fast Quenching, Photosynthetic Control and PSII Photoinhibition Resulting in PSI Photoprotection

**FIGURE 2:**
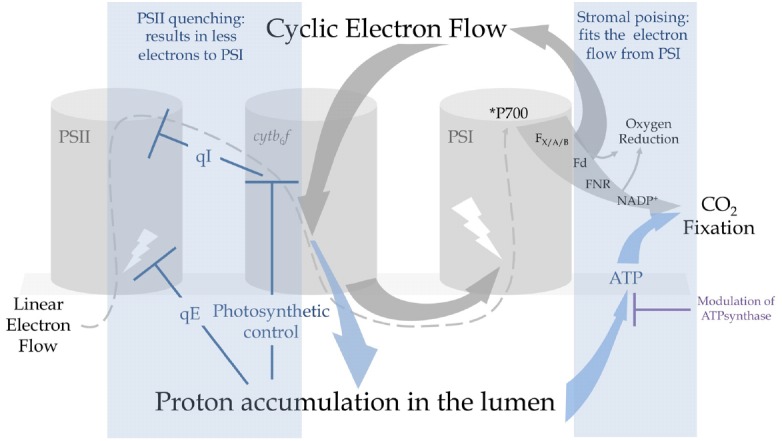
**Cyclic electron flow promotes proton accumulation in the lumen and triggers regulatory mechanisms that can protect PSI from photoinhibition.** Under constraining conditions, electrons are recycled from the acceptor-side of PSI by PGR5-CEF that results in a rapid acidification of the lumen. This promotes (i) the energy-dependent quenching of PSII antennas (qE) and (ii) photosynthetic control at the level of *cytb_6_f* that exerts reducing pressure on PSII to provoke a controlled photoinhibition (qI). These mechanisms result in a decrease of electron flow to PSI. Regulation of ATPsynthase conductivity by protons, electrochemical gradient partitioning and O_2_ photoreductive pathways produce ΔpH, producing ATP and contributing to the recycling of NADPH. Extra ATP produced by CEF is used by the Calvin-Benson-Bassham (CBB) cycle to assimilate CO_2_ and contributes to the regeneration of NADP^+^. Decreasing linear electron flow or increasing the sinks downstream of PSI avoids the over-reduction of ferredoxin (Fd) and PSI centers.

As already suggested (Sonoike, 2011), non-photochemical quenching (NPQ) of PSII avoids excessive electron flow to PSI *via* linear electron flow (LEF) to prevent photoinhibition. CEF limits electrons entering the thylakoid chain because it prompts both excitation-dependent quenching (qE) and indirectly PSII photoinhibition (qI), thus avoiding overflow to PSI. Acidification of the lumen triggers qE (Briantais et al., 1979) and occurs during CEF due to coupling of electron transfer and proton translocation in the cyt*b*_6_*f*. Since both LEF and CEF pass through cyt*b*_6_*f*, the exact contribution of CEF to the formation of a qE is hard to determine but an altered ability to develop qE is observed in *Crpgr5* and *Crpgrl1* strains (Tolleter et al., 2011; Dang et al., 2014; Johnson et al., 2014; Kukuczka et al., 2014) concomitantly with PSI photoinhibition (Dang et al., 2014; Johnson et al., 2014). This is also consistent both with the failure to acidify the lumen under short saturating illumination in *Atpgr5* plants (Suorsa et al., 2012) and reduced growth of *Crpgrl1* strains in fluctuating light (Dang et al., 2014). A recent report challenging the effects of rapid quenching of PSII in PSI photoprotection showed that an absence of qE (in *Atnpq4* mutants lacking the PsbS protein that induces qE in higher plants, Li et al., 2000) does not have a dramatic effect on P700 oxidation kinetics at any light regime as opposed to *Atpgr5* mutants where steady state oxidation of P700 is abolished (Tikkanen et al., 2015). Partial compensatory mechanisms may, however, act between CEF and qE as double mutant strain *Crpgrl1npq4*, (lacking both CEF and the LHCSR3 protein that acts as the activator for qE in *Chlamydomonas*, Peers et al., 2009), are particularly susceptible to PSI photoinhibition in comparison to the simple *Crpgrl1* mutant (Kukuczka et al., 2014; Bergner et al., 2015). This may coincide with a PSI-photoprotective role recently proposed for LHCSR3 via its association with the PSI antenna system under “state 2 conditions” (Allorent et al., 2013; Bergner et al., 2015) but here LHCSR3-dependent quenching of LHCIs and/or PSI-bound LHCIIs has not been strictly established. The argument against would be that the quenching of PSI antenna is irrelevant given the harmlessness of *P700 and furthermore photo-oxidation events at PSI (measured on isolated complexes *in vitro*) have been shown to take place after photoinhibition is completed, chlorophyll oxidation being preceded by irreversible carotenoid oxidation (Santabarbara, 2006). For now, the literature would suggest that qE is a first level of photoprotection in photoinhibitory conditions and rapidly protects not only PSII but also PSI by reducing electron flow (Figure 2).

Aside from ATP production and qE, high pmf downregulates LEF (Rumberg et al., 1968) at the site of PQH_2_ oxidation: originally called “back pressure,” (Stiehl and Witt, 1969), and now known as “photosynthetic control.” Comparison of the *Cr*Δ*rbcL* mutant against the double mutant *Cr*Δ*rbcL pgr5* or wildtype (WT) clearly shows the effects of photosynthetic control imposed by CEF witnessed by a strong increase in chlorophyll fluorescence in the single mutant indicative of a gradually decreasing electron flow, while the double mutant has fluorescence kinetics that resemble the WT (Johnson et al., 2014). The mutant lacking RuBisCO is an important genetic tool to observe the effects of an absence of CO_2_ fixation on CEF because it is difficult to modulate CO_2_ much below atmospheric concentrations in *Chlamydomonas* due to the efficiency of the carbon concentrating mechanism (CCM): thus this double mutant gives us a window on the mechanisms of CEF as if the WT were under strong CO_2_-limited conditions. As important as qE in fluctuating light, photosynthetic control is established when a rapid response to severe acceptor side limiting conditions is required to buffer a sudden burst of electron flow toward PSI (Suorsa et al., 2012). Moreover, while qE is not constitutive but inducible in *Chlamydomonas*, on the contrary to plants (Peers et al., 2009), photosynthetic control is likely to be crucial in the very first hours of exposition to drastic conditions. As a secondary consequence of photosynthetic control, reducing pressure increases on the Q_*B*_ site of PSII so that PSII centers remain in a closed state longer. Overexcited chlorophyll (^3^P680) activates O_2_ into ^1^O_2_ triggering photoinhibition of PSII (reviewed, in Sonoike, 2011; Murata et al., 2012). Highlighting the key role of pmf in control of linear electron transfer, PSI was shown more susceptible to photoinhibition than PSII in nigericin-infiltrated leaves where control at the level of *cytb*_6_*f* could not develop (Joliot and Johnson, 2011). Moreover, photoinhibition of PSI in *Atpgr5* could be avoided by modulating PSII turnover with the addition of the protein translation inhibitor lincomycin (Tikkanen et al., 2014). Thus, PSII photoinhibition mediated by photosynthetic control is a secondary level of photoprotection in drastic photoinhibitory conditions that exceed qE dissipation capacity: it indirectly but effectively acts as a shunt to avoid sustained PSI acceptor-side limitations (Figure 2).

In *Chlamydomonas*, the *pgr5* mutation combined with an absence of chloroplast ATPsynthase results in a less photosensitive phenotype than the ATPase mutant alone, where light sensitivity has been attributed to luminal over-acidification (Johnson et al., 2014). This observation shows that photosynthetic control relying on CEF actively contributes to decreasing the luminal pH and supports previous work (Rott et al., 2011). In higher plants, triggering of low luminal pH has also been correlated with changes in conductivity of the ATPsynthase to protons (Kanazawa and Kramer, 2002) and also to partitioning of the proton motive force between its osmotic (or concentration gradient, ΔpH) and electrical (ΔΨ) component (Avenson et al., 2005). ATPsynthase conductivity to protons is increased in *Atpgr5* (Avenson et al., 2005; Wang et al., 2015) with similar observations seen in knocked-down *PGR5* rice lines (Nishikawa et al., 2012). This may be ruled by the concentration of substrate for ATP production, i.e., ADP and phosphate (P_*i*_): in spinach thylakoids, artificially decreasing P_*i*_ levels resulted in lower ATPase conductivity and a lower luminal pH, thus promoting qE (Takizawa et al., 2008). These observations show the metabolic interconnections between ATP, CEF and ATPsynthase. As already suggested (Shikanai, 2014), further studies should be done to explain the acceptor-side limitation occurring in strains affected in PGR5-CEF in the light of the scenario proposed by Kramer and coworkers for qE regulation.

## O_2_ Reduction Works with CEF in Dissipating Excessive Electron Flow

A number of recent observations have shown the direct interaction of CEF with chloroplast metabolism because CEF regulates acceptor-side limitation in the absence of reactions for consumption of NADPH. Rubisco-less mutants but also CBB cycle mutants and those affected in starch metabolism show a strong increase in CEF or in CEF-dependent photosynthetic control that results in a repressed rate of LEF (Livingston et al., 2010; Johnson and Alric, 2012; Johnson et al., 2014; Krishnan et al., 2015). When the CBB cycle is an insufficient sink for reducing power, O_2_ photoreduction pathways may work in conjunction with CEF to protect PSI. On the other hand, under non-acceptor side limited conditions (steady state high light and/or high CO_2_) an absence of CEF in *Crpgrl1* did not result in photoinhibition to PSI and this capacity to acclimate was shown to be due to a sustained dependence on O_2_ photoreduction pathways (Dang et al., 2014). While photorespiration is a minimal process in green algae due to the CCM, other important sinks exist for reducing equivalents downstream of PSI that terminate on O_2_. These include: (i) export of reducing power to the respiratory chain to stimulate oxidative phosphorylation in the mitochondria, (ii) ROS-producing (“Mehler”) reactions with a concomitant increase in detoxifying enzymes and (iii) ROS-independent (“Mehler-like”) NADPH:O_2_ oxidoreduction probably by flavodiiron proteins (FLV; Peltier et al., 2010). Mechanisms (ii) and (iii) dually generate proton gradient and thus ATP (Forti and Elli, 1995, 1996) and regenerate NADP^+^ thus avoiding PSI acceptor-side limitations, and over-expression of FLV proteins 1 and 3 in cyanobacteria have been observed to stabilize PSI under fluctuating light (Allahverdiyeva et al., 2013). Mechanism (i) mitochondrial cooperation, also generates ATP but the ATP is probably not reshuttled back into the chloroplast, its major role would be thus to regenerate oxidized NADP^+^ (Figure 2).

Radmer and Kok (1976) first observed the potential for O_2_ to replace CO_2_ fixation during a light-to-dark transition or in the presence of CBB cycle inhibitors. The role of such an acceptor side activity within the chloroplast such as Mehler (O_2_ reduction) or hydrogenase (H^+^ reduction) would enable *Chlamydomonas* cells to reoxidise the electron transport chain in the light, convincingly shown after anaerobic incubation (Forti et al., 2005; Ghysels et al., 2013). In the *Crpgr5* Δ*rbcL*, lacking both CO_2_ fixation and CEF, O_2_ photoreduction rates can completely compensate for CO_2_ fixation resulting in WT O_2_ evolution levels (Johnson et al., 2014). Similarly, in a detached leaf assay addition of antimycin A provokes both production of H_2_O_2_ and a strong sustained malate dehydrogenase activity resulting in high rates of mitochondrial O_2_ uptake (Fridlyand et al., 1998). While very removed from the steady-state metabolic flow observed in WT strains under standard conditions, these experimental observations provide us with the maximal rates for the different pathways, and suggest possible compensatory reactions. It would appear that CEF down regulates ATP-independent O_2_ reducing pathways and up regulates ATP-dependent CO_2_ reduction by CBB cycle. Therefore, CEF can be seen as limiting ROS production under acceptor side limitations. Furthermore, it has been suggested that an interplay between CEF and O_2_ photoreduction acts as a buffer to poise electron flow toward carbon fixation (Backhausen et al., 2000). The action of H_2_O_2_ as an activator of NDH CEF in *Arabidopsis* provides further evidence that O_2_ photoreduction pathways and CEF are working in tandem (Strand et al., 2015). The model that emerges is that regulation of temporary excesses of reductant at the acceptor side of PSI is controlled by an interplay between CEF, the Mehler reaction, FLV proteins and the malate valve with another level of control exerted by redox regulators such as thioredoxins (Scheibe and Dietz, 2012). These pathways likely form a set of communicating reactions that can rebalance NADPH/NADP^+^ ratios and avoid PSI photoinhibition.

## Concluding Remarks

While the study of mutants reveals to us the limitations of a system, the complete photosynthetic apparatus is perfectly able to acclimate to both light and changing redox conditions with CEF and its protective role over PSI placed centrally as a regulator of this flexibility. Further understanding of PSI photoinhibition, proposed to be a major determinant in crop productivity (Tikkanen et al., 2014), may allow the rational modification of photosynthesis to improve the efficiency of plant crops and the production of renewable algal biomass.

### Conflict of Interest Statement

The authors declare that the research was conducted in the absence of any commercial or financial relationships that could be construed as a potential conflict of interest.
